# Asymptomatic ureteral metastasis of chromophobe renal cell carcinoma after radical nephrectomy: A case report and review of literature

**DOI:** 10.1016/j.ijscr.2024.109907

**Published:** 2024-06-12

**Authors:** Farhood Khaleghi mehr, Nasrollah Abian, Maryam Abolhasani, Yasaman Moradi

**Affiliations:** a Department of Urology, Hasheminejad Kidney Center, School of Medicine, Iran University of Medical Sciences, Tehran, Iran; b Department of Urology, 5Azar Hospital, School of Medicine, Golestan University of Medical Sciences and Health Services, Gorgan, Iran; c Department of Pathology, Hasheminejad Kidney Center, School of Medicine, Iran University of Medical Sciences, Tehran, Iran

**Keywords:** Chromophobe RCC, Metastasis, Renal cell carcinoma, Ureteral stump, Asymptomatic

## Abstract

**Introduction:**

Standard treatment for renal cell carcinomas (RCCs) is radical/partial nephrectomy and unlike upper urothelial carcinoma, complete ureteral removal is not necessary nor is advised in RCCs as tumor recurrence in ureteral remnant has scarcely been reported. Here, we present a rare case of chromophobe RCC (ChRCC) metastasis in remnant ureter following radical nephrectomy and perform a literature review in this regard.

**Case presentation:**

A 66-year-old man presented with a CT scan–as a surveillance protocol imaging- showing a mass in ipsilateral remnant ureter 9 months after radical nephrectomy due to ChRCC while being completely asymptomatic.

Cystoscopy revealed a polypoid mass protruding from right ureterovesical junction and transurethral resection of tumor revealed it to be a ChRCC. Distal ureterectomy was performed confirming pathology without any lymph node involvement.

12 months after ureterectomy, he remained asymptomatic with no sign of metastasis or recurrence in his follow up CT scan.

**Discussion:**

RCC metastasis to distal ureter after radical nephrectomy has been rarely reported and only 2 cases of them were ChRCC. Gross hematuria has been the main presentation of such disease. However, our case was completely asymptomatic, highlighting necessity of surveillance imaging.

No specific treatment guideline exists for such presentation but tumor resection has been the most common treatment modality.

**Conclusion:**

Metachronous RCC metastasis may occur in remnant ureter which can be completely asymptomatic, highlighting necessity of surveillance imaging and reviewing them meticulously. Surgical resection of the metastasis by distal ureterectomy seems to be the best treatment option.

## Highlights

• Renal cell carcinoma may metastasize to ureter after radical nephrectomy.
• This phenomenon might be completely asymptomatic.
• Ureterectomy seems to be the most appropriate treatment in this situation.
• Distant metastasis or recurrence in bladder has been reported after ureterectomy.
• Long term following up after ureterectomy with CT and cystoscopy is logical.

## 1 Introduction

Standard treatment for renal cell carcinomas (RCCs) is radical/partial nephrectomy [1]; complete ureteral removal (particularly ureteral stump) is not necessary nor is advised as tumor metastasis in remnant ureter is not expected in RCCs. However, about 60 cases of RCC in English literature have been reported to metastasize to remnant ureter; of them only 2 cases were chromophobe RCC [[2], [3], [4]] which is in proportion with the rarity of such pathology itself: only 5 % of all RCCs are chromophobe one [5]. Here, we present the third case of chromophobe RCC metastasis in remnant ureter following radical nephrectomy and perform a literature review in this regard.

## 2 Case presentation

**Fig. 1:**
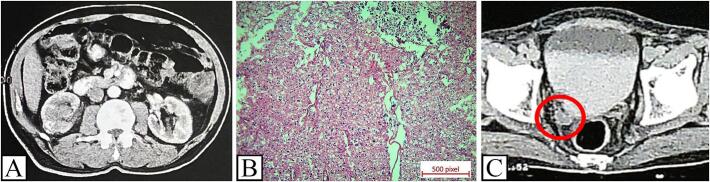
A. Abdominopelvic CT scan showing 4 cm enhancing solid mass in middle part of right kidney with extension into renal sinus. B. Pathology of radical nephrectomy specimen. H&E stained sections show chromophobe RCC with diffuse sheets of tumoral cells with eosinophilic nuclei, perinuclear halo and raisin like nuclei (×20). C. Follow up CT scan showing dilation of right distal ureter with a 22 mm mass at distal ureter and right ureterovesical junction (red circle). (For interpretation of the references to colour in this figure legend, the reader is referred to the web version of this article.)

A 66-year-old man presented with right flank pain and gross hematuria. He was under medical treatment for diabetes and hypertension while he mentioned no history of smoking or opium addiction. Serum creatinine level was normal. CT scan showed a 4 cm enhancing solid mass in middle part of right kidney with extension into renal sinus in favor of RCC or less probably upper tract urothelial carcinoma (Fig. 1A). Approaching gross hematuria, cystoscopy was normal and urine cytology was negative for malignancy. Thus, open right radical nephrectomy and lymphadenectomy through flank incision was performed and ureterectomy was deferred until reviewing the pathology.

The pathology report was chromophobe RCC (Fig. 1B) with tumor extension into renal sinus, free pelvicalyceal system, normal surgical margins including ureteral margin, no rhabdoid or sarcomatoid features, and no lymph node involvement (equal to pT3aN0M0 according to TNM staging).

**Fig. 2:**
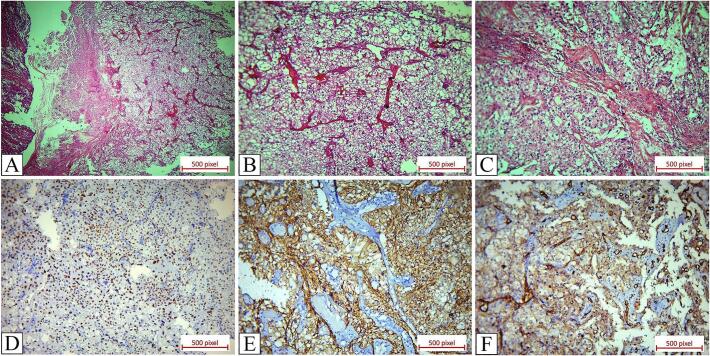
Pathology specimen of transurethral resection of protruding tumor from ureterovesical junction. A. H&E sections show sheets of epithelial tumoral cells with abundant cytoplasms (×10). B, C. H&E sections show chromophobe RCC with sheets of tumoral cells with abundant eosinophilic cytoplasms, distinct cell borders, perinuclear halo and raisin like nuclei (×20). D. Positive staining for PAX 8. E. Positive staining for E-Cadherin. F. Positive staining for C-Kit.

3 months after surgery, abdominopelvic CT scan was performed which was normal. 6 months later follow up chest and abdominopelvic CT scan showed dilation of right distal ureter with multiple masses up to 25 mm at distal ureter and right ureterovesical junction (Fig. 1C). Noticeably, the patient had neither urinary symptoms nor hematuria. Cystoscopy showed a polypoid mass protruding from right ureterovesical junction. Transurethral resection of tumor was performed. The hematoxylin and eosin sections revealed a tumor with morphological features of chromophobe RCC. The immunohistochemistry staining was positive for PAX 8, E-Cadherin, and C-Kit which confirmed the diagnosis of chromophobe RCC and were negative for CK 7, CK 20, P 63, GATA3, and thrombomodulin that ruled out the diagnosis of urothelial carcinoma and also negative for CD 10 that excluded the possibility of clear cell RCC (Fig. 2). The tumor involved lamina propria without muscular involvement (pT1).

**Fig. 3:**
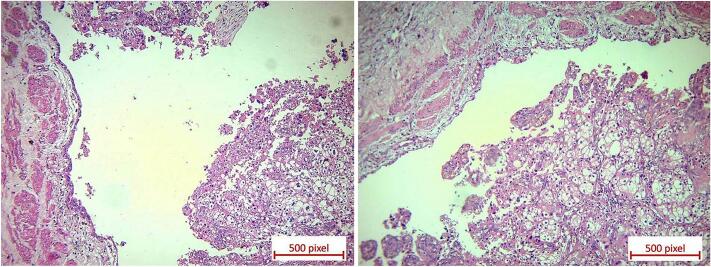
H&E stained sections of chromophobe RCC in lumen of right ureter showing same characteristics as primary tumor.

Therefore, right distal ureterectomy with bladder cuff excision and pelvic lymphadenectomy was performed. Pathology report confirmed multifocal involvement of ureter by chromophobe RCC with the largest tumor size of 2.5 cm while bladder cuff was free from tumor (Fig. 3). Twenty-three lymph nodes from pelvic region were also dissected which showed no tumoral involvement.

One year after the last surgery, he remains symptom free and his follow up cystoscopy and CT scan are normal. Our case report has been reported in line with the SCARE criteria [6].

## 3 Discussion

Radical nephrectomy (RN) is still the method of choice in advanced RCCs such as presence of tumor thrombosis or invasion to renal sinus [1]. While “RCC is well known for its unpredictable presentation, mode of spread, and tendency to early metastasis” [7], it is not common nor anticipated for RCC to metastasize to distal ureter; hence, ureterectomy is not advocated during RN. In fact, 61 cases (including our case) of RCC metastasis to distal ureter has been reported in English literature [2,3]. Clear cell RCC was the most common pathology while only 2 cases of chromophobe RCC have been reported [3,4]. We thereby report the third case of metachronous ipsilateral distal ureter metastasis of chromophobe RCC which unlike previous cases presented asymptomatically. The 3 cases of chromophobe RCC with metachronous metastasis to ipsilateral ureter are summarized in Table 1.

**Table 1:** Summary of cases of ipsilateral ureteral metastasis after radical nephrectomy due to chromophobe RCC.

Author/year	Age (years)/gender/side	Pathological characteristics of first tumor			Surgical margin after radical nephrect-omy	Time to diagnosis of ureteral metastasis after first surgery	Presentation of ureteral metastasis	Diagnostic modalities	Metastasis to other sites	Single ureteral metastasis or multiple ones	Treatment	Pathology of second surgery	Follow up
		TNM stage/maximum tumor dimeter	Invasion to pelvicalyceal system	Lymph node involvement/vascular invasion									
Macleod et al./2015 [4]	43/male/left	Not directly mentioned/5 cm	Yes (“tumor grew into the renal pelvis”)	No	Free	8 years	Gross hematuria	CT scan + retrograde left ureterogram + Ureteroscopy	None	Multiple ureteral metastases along the ureter	Open left ureterectomy	Islands and nests of tumor in favor of T2 chromophobe RCC	18 months, free from tumor
Chalokia et al./2021 [3]	50/female/right	pT2N0M0/not specified	No	No	Free	8 years	Gross hematuria	CT scan + Cystoscopy + transurethral resection	None	Single mass at ureterovesical junction	Robotic-assisted right ureteric stump excision along with bladder cuff	Chromophobe renal cell carcinoma with negative margins	60 months, free from tumor
Khaleghi Mehr et al./2024	60/male/right	pT3aN0M0/4 cm	No (free pelvicalyceal system despite invasion to renal sinus)	No	Free	9 months	No clinical presentation	CT scan (as surveillance protocol) + Cystoscopy + transurethral resection	None	Multiple ureteral metastases along the ureter	Open right distal ureterectomy with bladder cuff excision and pelvic lymphadenectomy	Multifocal involvement of ureter by chromophobe RCC	12 months, free from tumor

The time to metastasis has been reported from few months up to 12 years after surgery [8]. In our case metastasis happened within 9 months after surgery. Several hypotheses have been proposed regarding mechanism of these metastases including retrograde tumor cell spread from renal veins or lymphatic vessels, hematogenous metastasis, or direct intraluminal tumor cell spillage into ureter before and during RN [2,8]. Regarding our case, as primary tumor didn't invade pelvicalyceal system according to first pathology report, tumor cell spillage and seeding is less probable; thus, spread through blood or lymphatic vessels is the possible mechanism for metastasis. However, lack of any pathologic lymph nodes after second surgery reduces the possibility of lymphatic route spread, leaving metastasis through blood vessels as the best explanation in the presenting case.

However, it would be difficult to rule out direct tumor cell spillage as it seems to be a quite convincing mechanism for ipsilateral ureteral metastasis after RN. We thought if this mechanism was the main route of metastasis, such cases would be more common in RCCs invading to pelvicalyceal system. Therefore, we reviewed all reported cases of ipsilateral ureteral metastasis after RN from 2 decades ago till present time and surprisingly found that in only 1 out of 7 cases invasion to renal pelvis was seen [4]. Concordantly, systematic routes of metastasis i.e., through vessels rather than direct tumor cell spillage are more likely to be the best explanation for this phenomenon.

What makes our case special is the lack of symptoms at the time of metastasis. While most common presentation in reported cases was gross hematuria [[2], [3], [4],^7^,^8^], our patient had neither hematuria nor any urinary symptoms; and diagnosis of metachronous metastasis to ipsilateral ureteral stump was made through rigorous follow up imaging, highlighting the necessity of surveillance protocols after radical nephrectomy.

Tumor removal through surgery has been the main treatment. Metastasectomy was the most common management in almost all reported patients [[2], [3], [4],^7^,^8^]. Just similar to previous cases, our patient underwent distal ureterectomy and bladder cuff excision.

Prognosis of such cases is not well documented. Uneventful follow up has been reported for up to 5 years [2,3] although data regarding specific follow up protocols are scarce. Both distant metastasis (e.g. lung) and tumor recurrence within bladder have been reported after metastasectomy of ureteral metastasis [2,9]. Thus, it is wise to consider routine cystoscopy along with CT scan as surveillance protocol in such cases. Follow up cystoscopy and CT scan have been normal in our patient and he has remained tumor free 12 months after distal ureterectomy and bladder cuff excision.

## 4 Conclusion

RCC may metastasize to most unexpected sites long after its removal. Remnant ureter after radical nephrectomy is a potential site of metastasis. As in our case, this metastasis can be completely asymptomatic, highlighting necessity of rigorous surveillance imaging and reviewing them meticulously. Surgical resection of the metastasis by distal ureterectomy seems to be the best treatment option.

## Abbreviations

RCC: renal cell carcinoma
ChRCC: chromophobe RCC
RN: radical nephrectomy

## Consent

Written informed consent was obtained from the patient for publication of this case report and accompanying images. A copy of the written consent is available for review by the Editor-in-Chief of this journal on request.

## Ethical approval

This case report is exempt from ethical approval due to the nature of the article (a retrospective description of a rare disease), as per the ethical review board at our institution.

## Funding

This research did not receive any specific grant from funding agencies in the public, commercial, or not-for-profit sectors.

## Author contribution

Study concept: Farhood Khaleghi mehr, Nasrollah Abian

Data collection: Farhood Khaleghi mehr, Nasrollah Abian

Data interpretation: Nasrollah Abian, Maryam Abolhasani, Yasaman Moradi

Writing the paper: Nasrollah Abian

Revision: Farhood Khaleghi mehr

## Guarantor

Nasrollah Abian, Farhood Khaleghi mehr.

## Research registration number

Our case report is not “First in Man”.

## Conflict of interest statement

The authors declare that there is no conflict of interests regarding the publication of this article.
